# Panel Dataset of Ethical Commitment Disclosures in Malaysia

**DOI:** 10.1016/j.dib.2020.105624

**Published:** 2020-04-28

**Authors:** Hafiza Aishah Hashim, Ahmad Firdhauz Zainul Abidin, Zalailah Salleh, S. Susela Devi

**Affiliations:** a Faculty of Business Economics and Social Development, Universiti Malaysia Terengganu, 21030, Kuala Nerus, Terengganu, Malaysia; b Sunway University, No. 5, Jalan Universiti, Bandar Sunway, 47500 Selangor Darul Ehsan, Malaysia

**Keywords:** Ethics, Whistleblowing, Code of ethics, Disclosures

## Abstract

Panel dataset in this article contains information on the ethical commitment disclosures of Malaysian publicly listed companies. The data presented is related to the research article entitled “Ethical Practice Disclosure of Malaysian Public Listed Companies” [1]. In examining the level of ethical commitment disclosures, content analysis is performed involving 1,115 annual reports for five year periods (2012 – 2016). The annual reports are gathered from Main Market of Bursa Malaysia website. Information on ethical commitment disclosures are extracted from the annual reports. The data are collected using Ethical Commitment Index (ECI) comprising six themes; corporate ethics values, action to promote ethics, whistle-blowing policy, code of ethics, sustainability practices, and ethics committee. This dataset is useful as an indicator of the companies’ ethical commitment reflecting ethical climate in Malaysian public listed companies.

Specifications Table

**Table:** 

Subject	Business, Management and Accounting (General)
Specific subject area	Business ethics
Type of data	Table Graph
How data were acquired	Companies’ annual report downloaded from Main market in Bursa Malaysia, Content analysis from companies’ annual reporting, Data gathered using extended Ethical Commitment Index [1].
Data format	Raw Filtered
Parameters for data collection	Non-financial companies listed in Main board of Bursa Malaysia. Companies with missing data and/or annual report are excluded.
Description of data collection	Content analysis of 223 companies’ annual reports for five years observations (2012-2016), total 1,115 companies annual report
Data source location	Malaysia
Data accessibility	With the article, and Repository: Mendeley Data https://data.mendeley.com/datasets/4pnd65dpnh/draft?a=8d4746a9-173b-4dc3-a2c4-4456f2f19be5
Related research article	Ahmad Firdhauz Zainul Abidin, Hafiza Aishah Hashim, Zalailah Saleh, S.Susela Devi, Ethical Practice Disclosure of Malaysian Public Listed Companies, in FGIC 2nd Conference on Governance and Integrity 2019, KnE Social Sciences, pages 1168–1201. DOI 10.18502/kss.v3i22.5119 [1]

## Value of the data

• The dataset provides preliminary data on various attributes of ethical commitment/practices disclosed by Malaysian companies.
• The dataset is useful for companies and policy makers to assess the level of ethical commitment disclosures of companies and implement action plans to improve the ethic practices.
• The dataset can be used as part of corporate governance framework and have a potential to be used toward different model in evaluating corporate values.

## 1 Data Description

**Fig. 1:**
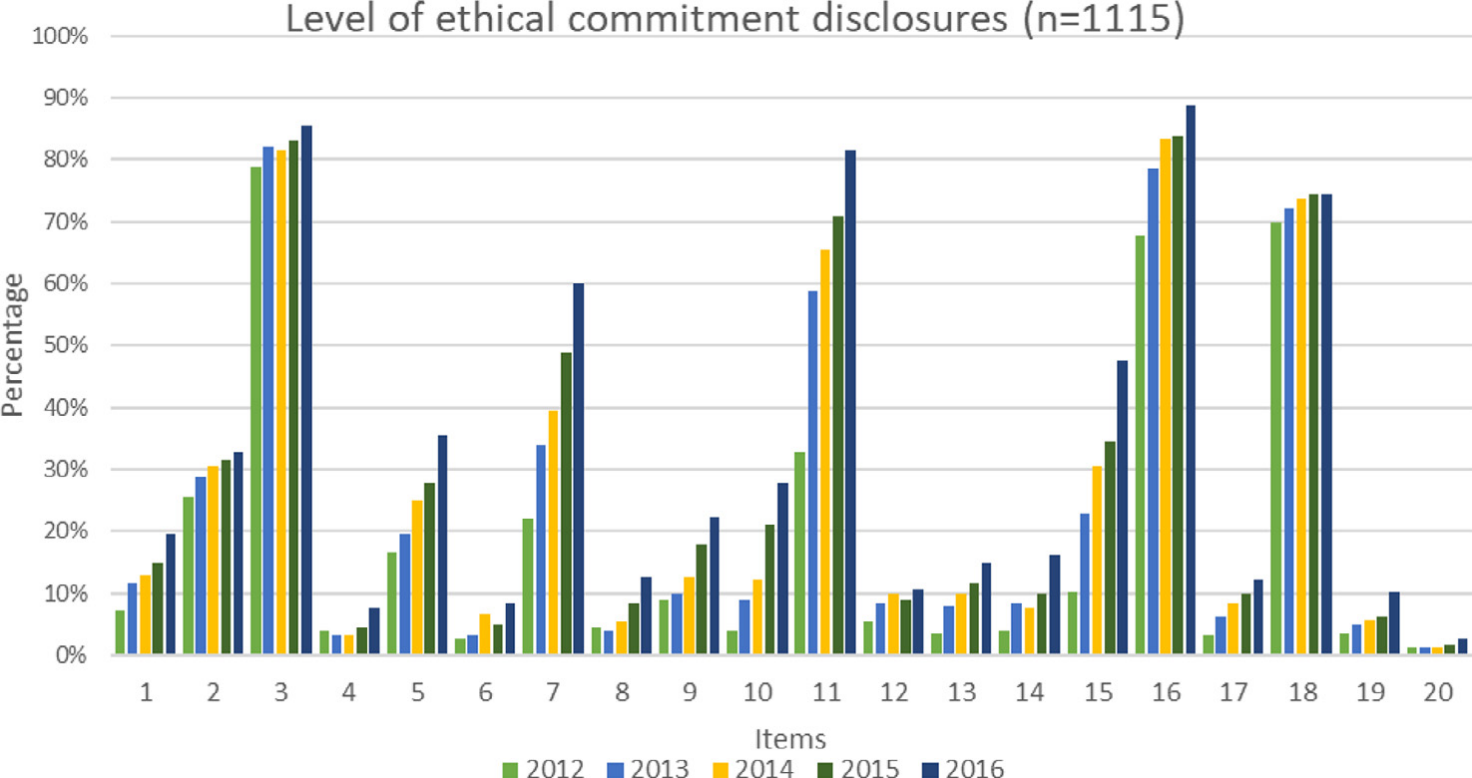
The level of ethical commitment disclosures (N=1115).

**Fig. 2:**
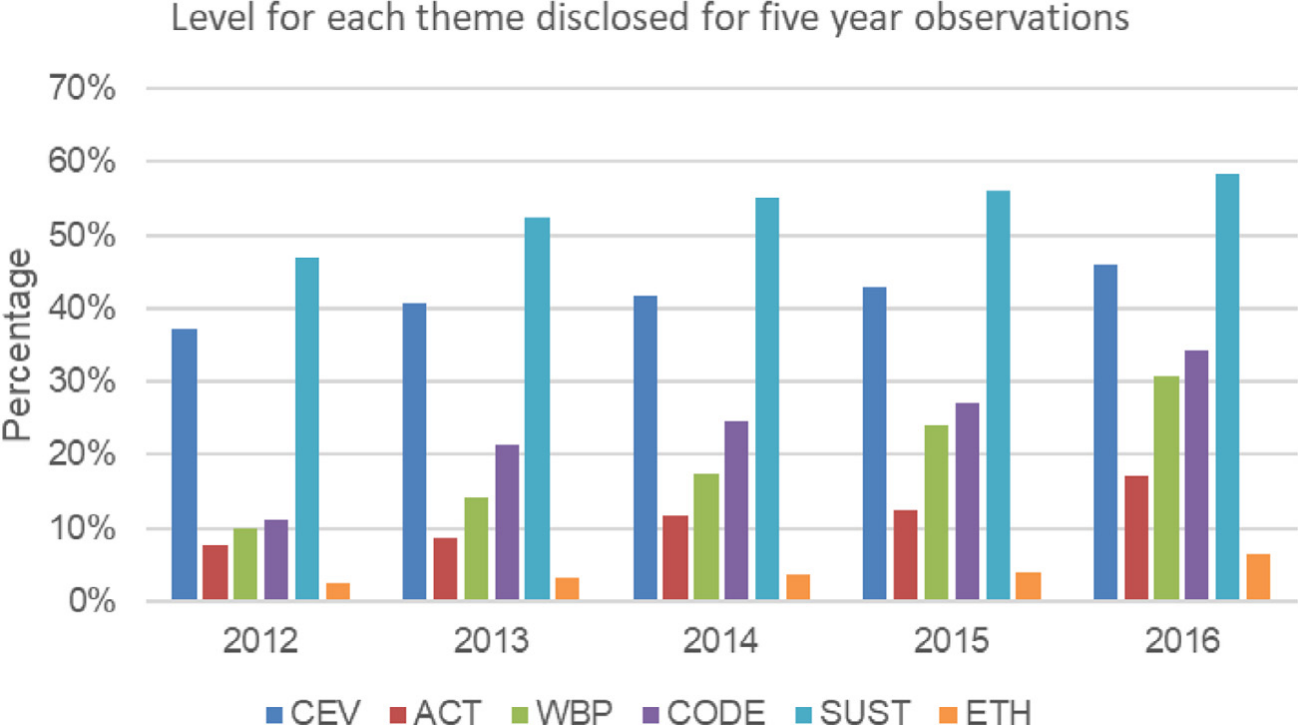
The level for each theme disclosed for five year observations.

The dataset contains 223 companies (1,115 observations) from twelve sector in Main Market of Bursa Malaysia listings as in Table 1. The study applied a systematic random sampling approach to select companies using Microsoft Excel to obtain a sample that appears to be representative of the population. Table 2 presents the original instrument to measure the ethical commitment originally developed by Choi and Jung [2]. Extended version of ECI are presented in Table 3 with modifications made by adding 9 additional items and aggregate all the items into six themes that resulted in final 20 items to be used in calculating ECI score. The index was modified and adapted to suit Malaysian corporate environment. Fig. 1 presents the level of ethical commitment disclosures based on the percentage of companies disclosing each items from ECI for five years’ observations (2012 to 2016). The level of each themes of ECI are presented in Fig. 2. In supplementary materials provided, the file is about the list of sample companies with their industry-sectors and binary score for each items, together with the total ECI scores for each company. Each sheets in the excel represent years.

**Table 1:** Descriptive statistics for company sector in Bursa Malaysia for year (2012 to 2016).

SECTOR BURSA	Freq. (companies)	Percent (%)	Cum.
Construction sector	60	5.38	5.38
Consumer products & services sector	240	21.52	26.91
Energy sector	45	4.04	30.94
Health care sector	35	3.14	34.08
Industrial products & services sector	360	32.29	66.37
Plantations	65	5.83	72.2
Property	140	12.56	84.75
Real estate	25	2.24	87
Technology	45	4.04	91.03
Telecommunications & media	30	2.69	93.72
Transportations & logistics sector	55	4.93	98.65
Utilities	15	1.35	100
**Total**	**1,115**	**100**

**Table 2:** Original ECI developed by Choi and Jung [2].

Description

1. Top managers of this company regularly emphasize the importance of business ethics
2. Ethical behaviour based on a formal business philosophy is the norm of this company
3. This company has a disciplinary system through which unethical behaviour is strictly punished
4. This company has a code of ethics
5. In this company, employees can report unethical conduct through an anonymous channel
6. In this company, ethics education, training, or workshops are in place to enhance business ethics of employees
7. This company regularly puts a significant portion of its profits toward philanthropy
8. This company has an independent ethics department and officers
9. In this company, employees can get help regarding business ethics through an ethics hotline or open communication channel
10. This company has an ethics committee
11. This company has an ethics evaluation system measured by an independent party from outside the company

**Table 3:** Extended ECI.

Themes	Items	Descriptions
CEV	1	Top managers of this company regularly emphasise on the importance of business ethics
	2	This company has ethics philosophies and ethical values
	3	This company is committed towards the highest standard of business practices
ACT	4	This company has a disciplinary system in which unethical behaviour is strictly punished
	5	This company provides training, workshops, and education-related ethics towards employees
	6	This company has employee appraisal programmes to promote ethical conduct
WBP	7	This company establishes the whistleblowing policies
	8	This company has an open communication channel for employees to assist with ethical issues
	9	This company has whistle-blower protections
	10	This company provides whistleblowing policies in the website
CODE	11	This company has formulated the code of ethics
	12	This company uses formal and informal methods to communicate the code of ethics
	13	This company has implemented a system to ensure the compliance of the code of ethics
	14	This company has revised its code of ethics periodically
	15	Code of ethics is available in company websites
SUST	16	This company is committed to sustainability practices
	17	This company regularly invested a significant portion of profits to philanthropy
	18	The sustainability practice report of this company is available in the website
ETH	19	This company has an ethics committee
	20	This company has an independent ethics department and officers

## 2 Experimental Design, Materials, and Methods

The dataset represents 223 companies randomly selected from Bursa Malaysia main board after excluding financial sectors and missing data and/or annual reports. The dataset consists of 1,115 companies for five year observations from year 2012 to year 2016. Table 1 shows the descriptive statistics for twelve companies’ sector involved (real estate, technology, telecommunications & media, transportations & logistics, utilities, energy, health care, constructions and plantation).

Table 2 presents the explanations of all themes and items of ethical commitment index. ECI previously developed by Choi and Jung [2] are extended by incorporating the perspective of Malaysian corporate environment to measure companies’ ethical commitment. The development of ECI was based on previous literatures [3], [4], [5] and MCCG recommendations. The basic of ECI was constructed based on formal methods such as code of ethics, ethics training, ethics evaluation, ethics committee and officers to promote ethics in company [3]. As there is no standard measure, ECI in Table 2 were extended by adding 9 items and aggregated each item into 6 themes (Table 3). The themes in the extended ECI include corporate ethics values and philosophy (CEV), actions to promote ethics and prevent unethical conduct (ACT), whistle-blowing policies (WBP), code of ethics (CODE), sustainability practices (SUST) and ethics committee (ETH). Data of each themes and items together with ECI score is provided in supplementary materials.

Binary scoring method is used to measure each item; whether disclosed or not. One (1) point is given for the disclosure of particular items in annual reports, and zero (0) point if no disclosure is made at all. Binary scoring was chosen due to the nature of the information gathered from each item in ECI that consists of six themes of ethics disclosures. Within each of the themes there are items that represent the detailed information of the themes, thus binary scoring allows information regarding ethics to be examined in depth [6]. In addition, this method allows determinations of companies into those that are committed toward ethics or not [7]. Data of ethical commitment disclosures are hand collected through content analysis.

## Conflict of Interest

The authors declare that they have no known competing financial interests or personal relationships which have, or could be perceived to have, influenced the work reported in this article.
